# Direct Writing of Quasi-Sinusoidal and Blazed Surface Relief Optical Transmission Gratings in Bi_12_GeO_20_, Er: LiNbO_3_ and Er: Fe: LiNbO_3_ Crystals by Nitrogen Ion Microbeams of 5 MeV and 10.5 MeV Energy

**DOI:** 10.3390/s25030804

**Published:** 2025-01-29

**Authors:** István Bányász, Gyula Nagy, Vladimir Havránek, Maria Cinta Pujol, Ágnes Nagyné Szokol, György Kármán, Robert Magnusson, István Rajta

**Affiliations:** 1 Department of Physics and Chemistry, Széchenyi István University, H-9026 Győr, Hungary; 2 Atomki, HUN-REN Institute for Nuclear Research, P.O. Box 51, H-4001 Debrecen, Hungary; gyulanagy@atomki.mta.hu (G.N.); rajta@atomki.hu (I.R.); 3 Nuclear Physics Institute AV CR, 250 68 Řež near Prague, Czech Republic; havranek@ujf.cas.cz; 4 Departament Química Física i Inorgànica, Universitat Rovira i Virgili, Campus Sescelades, E-43007 Tarragona, Spain; mariacinta.pujol@urv.cat; 5 HUN-REN Wigner Research Centre for Physics, P.O. Box 49, H-1525 Budapest, Hungary; szokol.agnes@wigner.hu (Á.N.S.); karman.gyorgy11@gmail.com (G.K.); 6 Department of Electrical Engineering, University of Texas at Arlington, Arlington, TX 76019, USA; magnusson@uta.edu

**Keywords:** transmission optical grating, surface relief, sinusoidal grating, blazed grating, ion microbeam, ion beam irradiation, grayscale lithography

## Abstract

High diffraction efficiency optical transmission gratings with quasi-sinusoidal and saw-tooth surface relief profiles were fabricated in Bi_12_GeO_20_, Er: LiNbO_3_ and Er: Fe: LiNbO_3_ crystals by ion beam implantation. The gratings were directly written by nitrogen ion microbeams at energies of 5 MeV and 10.5 MeV. The finest grating constant was 4 μm. Grating constants for the majority of the gratings were 16 μm. The highest amplitudes of the gratings reached 1600 nm. The highest first-order diffraction efficiency obtained in a sinusoidal grating was 25%, close to the theoretical maximum of 33%. The highest first-order diffraction efficiency of a blazed grating was also 25%, without Littrow optimization. Such gratings can be incorporated into integrated optical biosensors.

## 1. Introduction

The use of ion beam implantation for material modification in microelectronics began over 60 years ago [1]. These applications aimed at the precise control of the electric properties of semiconductor devices. The first use of ion beam implantation for the fabrication of planar optical waveguides was reported in 1967 [2].

High-energy ions modify the optical parameters of the target materials, not only their electronic properties. The ions interact with both the electrons and the nuclei of the target atoms through their trajectory in the target from the surface to the stopping point. In the case of optical crystals and glasses, the absorption and index of refraction of the material are mainly changed via the creation of color centers (defects) and amorphization of the target. Depending on the composition of the target material and the species, and the energy and fluence of the implanted ions, considerable volume changes (swelling or compaction) can also be produced in the target. The optical effects of ion implantation were extensively treated in the book of Townsend and co-authors [3]. Schineller and co-authors first used ion beam implantation for the fabrication planar optical waveguides [2].

Later, much progress was made in the field of the fabrication of optical elements using ion beam technics. These techniques are mainly used for the fabrication of planar and channel waveguides. For the fabrication of planar waveguides, homogeneous macroscopic ion beams are needed. As for channel waveguides, in early research, implantation by macrobeams through various masks was used. Another method, i.e., direct writing with ion microbeams, was introduced more recently. Regarding the quality of ion-beam-implanted optical waveguides, their parameters are similar to or even better than those which are produced by traditional methods, e.g., ion exchange or laser writing. Recent progress in this field is summarized in a review by Feng Chen [4].

Optical gratings have also been fabricated using both macrobeam implantation through a mask or focused ion beam writing since the late 1970s. An acoustic surface wave resonator was fabricated by Hartemann [5]. Those authors implanted a 100 keV He^+^ beam at a fluence of 1.5 × 10^16^ ion/cm^2^ through a mask into a quartz substrate. With a grating of 80 LP/mm, a diffraction efficiency of 99% was obtained at a frequency of 125 MHz. Garvin and co-workers reported on diffraction gratings with a grating constant of 370 nm, which were obtained using a holographically recorded photoresist mask and ion beam milling with an Ar^+^ ion beam in the energy range of 3–10 keV in a GaAs sample [6].

The successful fabrication of grating couplers of very low diffraction efficiencies (0.01–0.05%) in optical waveguides in a LiNbO_3_ and Corning glass samples was reported by Kurmer and Tang. Those authors implanted B^+^ and N^+^ ions of energies of 200 and 170 keV at fluences of around 10^16^ ion/cm^2^ in the targets. [7].

The fabrication of ion beam implanted gratings in silicon solar cells was reported by Hwang and co-authors [8], who used boron ions in the energy range of 20–180 keV and fluences between 10^11^ and 10^15^ ion/cm^2^. They combined various fabrication steps, like chemical etching, with the implantation. Dammann gratings with grating constants of 125 μm in both directions were produced in these experiments.

A focused ion beam (FIB) technique was used for the production of a phase mask, intended for the photo imprinting of fiber Bragg gratings, by Erickson et al. The target was fused silica, and a 200 keV Si^++^ beam of 100 nm diameter was used. The sample was subsequently wet etched in an HF solution [9]. Orth et al. published various articles on the focused ion beam fabrication of distributed feedback lasers in GaInAs/GaAs [10,11,12]. Various integrated optical elements, like channel waveguides, grating couplers, Mach–Zehnder interferometers, ring resonators, and directional couplers, were fabricated in silica substrates via FIB implantation with germanium ions by Yu et al. [13].

Roberts and von Bibra were the first to successfully use a focused proton beam in the energy range of 1–3 MeV to fabricate low-loss buried channel waveguides in fused silica in the 1990s [14]. Three-dimensional microstructures were designed and fabricated by Schrempel and Witthuhn [15], who used 1.8 MeV proton beams with widths of 10 and 50 μm. This remained a multi-step process, as ion microbeam writing was followed by the selective etching of the proton-beam-irradiated regions of the sample.

A review article on early progress in proton beam writing in microphotonics was published by Bettiol et al. [16]. They successfully applied direct proton beam writing to the fabrication of channel waveguides, optical gratings, microlens arrays, and colloid crystal templates. They also produced optical gratings in poly (methyl methacrylate) (PMMA) samples by adding selective chemical etching. Gratings of small lateral dimensions (100 μm × 30 μm), with grating constants in the range of 600–1200 nm, were produced.

High aspect ratio surface relief structures were written using a 3 MeV proton microbeams with 2–3 μm × 2–3 μm lateral dimensions by Glass et al. [17]. Those authors applied a subsequent chemical etching process to complete the fabrication of the microstructures in PMMA and SU-8 photoresist.

A focused proton beam of 2 MeV energy and diameter less than 2 μm was used by Huszank et al. to fabricate optical diffraction gratings and Fresnel zone plates in poly(dimethylsiloxane) (PDMS) [18]. Gratings constants were between 20 and 50 μm.

Romanenko et al. successfully fabricated optical gratings in PMMA polymers using a 10 MeV O^4+^ ion microbeam [19] and 2 MeV proton microbeam [20].

The main problem with the techniques just reviewed was that the resulting ion beam fabricated optical gratings were of low diffraction efficiency, and the profiles of the individual grating lines were not easy to control.

Accordingly, the design and fabrication of high-spatial frequency ion beam implanted optical gratings in glass samples with photoresist masks, using light and medium-mass ions, were performed by Bányász et al. [21]. They produced optical transmission gratings with first-order diffraction efficiencies up to 18% when implanted with 1.6 MeV N^+^ ions. They used light microscopy with high-power objectives, with and without immersion, and electron microscopy to study the implanted gratings. According to their results, it was the ion beam induced surface relief modulation that produced the greatest part of the optical path modulation across the gratings, namely, around 80%. Refractive index changes contributed only about 20% to the optical path modulation [22]. A quasi sinusoidal surface relief profile was obtained for the finest gratings with period of 4 μm. This fact could be explained by the lateral straggling of the implanted ions which was commensurate with small periods at the given parameters of the ion beam and the target [22].

García et al. recently reported preliminary results of direct writing of surface relief saw-tooth optical gratings on diamond using a microbeam of 9 MeV C^3+^ ions at fluences between 10^14^ ions/cm^2^ and 3 × 10^15^ ions/cm^2^ for use with soft X rays in a synchrotron [23]. Fabrication of a small grating with 12 blazed lines with a total width of 320 μm took a few hours. The amplitude of the fabricated grating was about 40% of the design amplitude. The profile of the ion beam implanted grating was sinusoidal instead of the designed saw-tooth profile. The authors attributed the deviations from the ideal profile to the elastic properties of the diamond target.

We reported successful fabrication of quasi-sinusoidal surface relief transmission gratings in IOG (a sodium-aluminophosphate laser glass, developed by Schott A.G.) and Pyrex [24]. Nitrogen and oxygen microbeams with energies of 5–6 MeV were used in the experiments. Higher-order diffraction efficiencies, and hence amplitudes of the higher harmonics of the grating profiles, were very low compared to the first-order ones. The highest amplitudes of the gratings exceeded 250 nm and the highest measured diffraction efficiency was 26%.

## 2. Materials and Methods

### 2.1. Design of the Gratings

The purpose of the research presented here was to produce high-spatial frequency surface relief optical gratings of quasi-sinusoidal and saw-tooth profiles having high diffraction efficiency in optical crystals. Based on our earlier results, we applied microbeams of medium-mass ions. It was the lateral dimensions of the ion microbeams that set the lower limit of the grating constant. In our earlier experiments in the fabrication of transmission optical gratings by ion beam implantation through masks, it was found that the gratings with periods of 4 μm exhibited a quasi-sinusoidal profile. That fact was attributed to the lateral straggling of the ion beam [21,22]. Based on those results, we expected that writing binary gratings with the microbeams used in our current experiments would also result in quasi sinusoidal grating profiles. Lateral dimensions of the ion microbeams used in the experiments were in the range of 1.8–2.4 μm. Hence, the lowest possible grating constant was about 4 μm.

**Figure 1:**
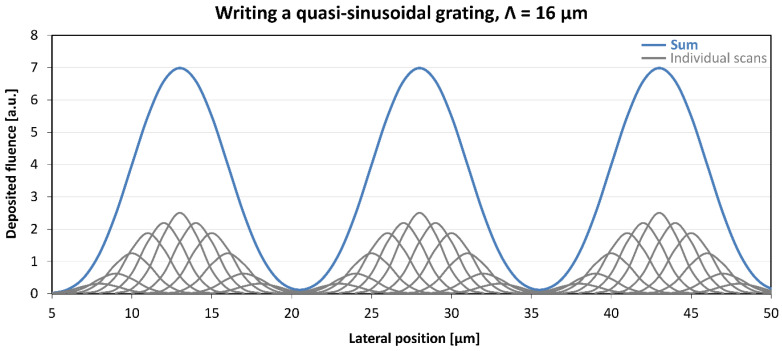
Total deposited fluence along of a sinusoidal grating of a grating constant of 16 μm. The profile of the ion microbeam was approximated by Gaussian with a FWHM of 3 μm. Fluence distributions of the individual scans are represented by gray lines. Individual scans were separated by 1 μm. Sum of the deposited fluence distributions of the individual scans is represented by the blue line. This figure was originally published in [24].

A method was developed for the fabrication of gratings with the ratio of the design grating constant to the ion beam width exceeding 2. Gratings with quasi-sinusoidal and saw-tooth profiles were designed and fabricated. It was expected that the effect of the ion microbeam on the target material would be proportional to the fluence. Evidently, this holds true only for an adequately chosen fluence range. Based also on our previous results, it was anticipated that this effect was mainly due the local swelling of the target surface under the microbeam. Besides that dominant effect, the high-energy ion microbeams induce changes under the target surface too, especially in changing its absorption and index of refraction. Based on the above assumption, we developed and successfully applied a procedure to produce surface relief gratings in optical materials [24]. The method was described in detail in that article. Here, we briefly summarize it, based on [24]. Each grating period was written by passing the microbeam multiple times. Fluence was spatially modulated to obtain a quasi-sinusoidal distribution of the total implanted fluence. An example of the planning of a sinusoidal grating is shown in Figure 1, taken from [24].

With the assumption that the height of the local surface swelling is linearly proportional to the locally deposited total fluence, one would expect that the profile of the surface relief gratings would be quasi-sinusoidal.

**Figure 2:**
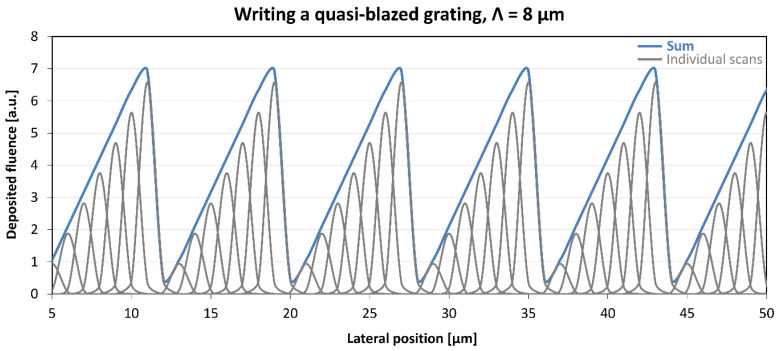
Distribution of the deposited fluence across a saw-tooth grating of a grating constant of 16 μm. The profile of the ion microbeam can be considered Gaussian with a FWHM of 1 μm. Fluence distributions of the individual scans are represented by gray lines. Lateral separation of the scans is 1 μm. Sum of the deposited fluence distributions of the individual scans is represented by the blue line.

The planning of a saw-tooth grating is presented in Figure 2. Similar to the design of the quasi-sinusoidal gratings, the saw-tooth profile was produced by performing a series of scans with the microbeam of Gaussian spatial profile and modulating the deposited fluence from scan to scan so that the resultant spatial distribution of the deposited fluence (the sum of the fluences deposited in the individual scans) approached a saw-tooth function.

### 2.2. Implantation of the Gratings

We designed and fabricated transmission optical gratings with sinusoidal and saw-tooth profiles in various optical crystals using high-energy nitrogen microbeams at the Tandetron laboratory of the UJF research institute in Řež, Czech Republic. The results presented here were obtained in the following materials: Sillenite type bismuth germanate crystal (Bi_12_GeO_20_), Er: LiNbO_3_, and Er: Fe: LiNbO_3_ crystals. All the crystal samples were fabricated at the Department of Crystal Physics of the HUN-REN Wigner Research Centre for Physics (Budapest, Hungary) by the Czochralski method. The LiNbO_3_ crystals were stoichiometric. The erbium concentration in the crystals was 0.5 atomic %. Fe concentration in the Er: Fe: LiNbO_3_ crystal was 0.6 atomic %. The experimental conditions of the various implantation experiments are presented in Table 1.

Numerous gratings were fabricated in Bi_12_GeO_20,_ Er: Fe: LiNbO_3_ and Er: LiNbO_3_ crystals. A grating constant of 16 μm was chosen for the greatest part of the implanted gratings. Given the lower limit to the lateral size of the ion microbeam, that value allowed for a sufficient number of scans for high-fidelity approximation of the desired grating profiles, as can be seen in Figure 1 and Figure 2.

Implantations were performed either with N^3+^ microbeams with an energy of 5 MeV or N^4+^ microbeams with an energy of 10.5 MeV. Based on earlier experiments, it was expected that implantation with the higher energy microbeam would greatly reduce the fluences necessary for the fabrication of the gratings [25]. At least two values of the implanted fluences were used for the fabrication of sinusoidal and blazed gratings at both energies. Ranges of the implanted fluences were chosen based on our previous experiments on the direct ion microbeam writing of channel waveguides in the same materials, e.g., in Bi_12_GeO_20_ [25]. While the formation of a surface relief structure (ridge) over the ion microbeam written channel waveguides was an unwanted secondary effect in those experiments, it became an excellent and controllable method for the fabrication of the gratings.

As stated earlier, a lower limit of the grating constant of the implanted gratings was imposed mainly by the lateral dimensions of the microbeam. The lowest value of the successfully fabricated grating period was 4 μm. However, detailed results only on gratings of a grating constant of 16 μm are presented in this article to establish quantitative relationships among the parameters of the ion microbeam implantation and those of the produced gratings.

### 2.3. SRIM Simulations

It is not easy to predict the effects of ion beam implantation in a target material. Those effects are determined in an intricate way by the composition and structure of the target material, the species of the implanted ions, their energy, the implanted fluence, and the current density (ion flux) [3]. If ion microbeams are used, the beam size also must be taken into account.

There are various computer codes for the simulation of the interaction of the implanted ions with the target material. The Stopping and Range of Ions in Matter (SRIM) code [26] was used to perform simulations for the actual experimental parameters to estimate the possible effects of ion implantation.

**Figure 3:**
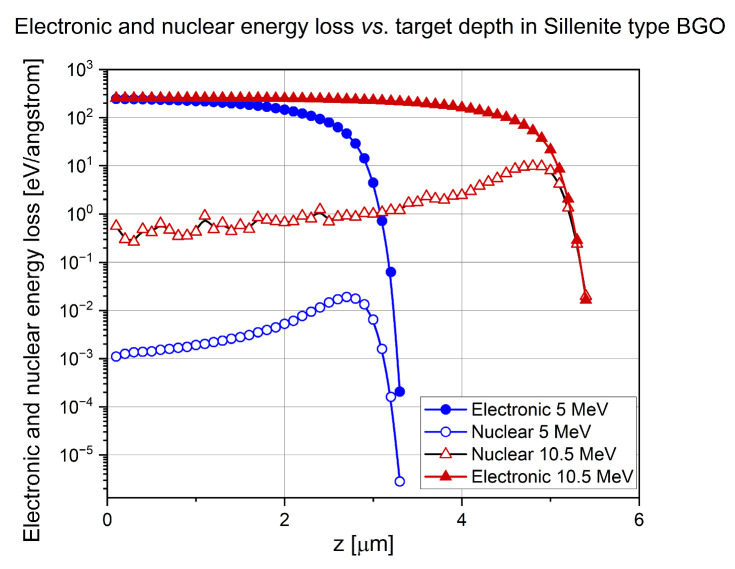
Electronic and nuclear energy loss of 5 MeV N^3+^ and 10.5 MeV N^4+^ ions in Bi_12_GeO_20_.

**Figure 4:**
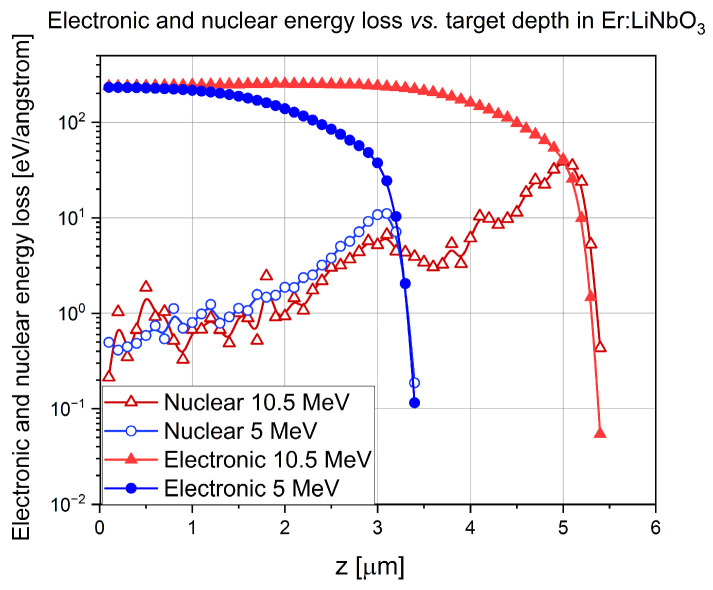
Electronic and nuclear energy loss of 5 MeV N^3+^ and 10.5 MeV N^4+^ ions in Er: LiNbO_3_.

Both the electronic and nuclear stopping powers for both energies of implanted ions and both target materials were calculated. The SRIM 2013 Pro version was used. The “Ion Distribution and Quick Calculation of Damage” option was chosen for the simulations. Mass densities of all the targets were taken from reliable literature instead of using the values provided by SRIM to avoid possible errors. There were 5000 incoming ions in each simulation. The results are presented in Figure 3 and Figure 4.

The maximum of the electronic energy loss was between 200 and 250 eV/angstrom for both 5 MeV and 10.5 MeV nitrogen ions, in both Bi_12_GeO_20_ and Er: LiNbO_3_ crystals. The width of the very broad and flat maximum of electronic energy loss vs. target depth depended almost linearly on ion energy, i.e., around 2 μm for the 5 MeV N^3+^ ions but around 4.5 μm for 10.5 MeV N^4+^ ions in both crystals at half of the maximum.

The height of the maximum of the nuclear energy loss vs. curve was about 0.02 and 10 eV/angstrom for the lower and higher energy nitrogen ions in Bi_12_GeO_20_. As for the Er: LiNbO_3_ crystal, the corresponding values were 10 and 40 eV/angstrom.

Bragg peak of 5 MeV N^3+^ ions was at 2.8 μm and that of 10.5 MeV was at 4.8 μm in Bi_12_GeO_20_. Bragg peak of 5 MeV N^3+^ ions was at 3 μm and that of 10.5 MeV was at 5 μm n Er: LiNbO_3_.

Structural and volume changes caused by ions of this mass and energy can be treated in the framework of the thermal spike model [26,27,28,29]. The main effect of the interaction of the ion microbeam with the target is mainly the local swelling of the target surface under the beam. This effect can be explained by the plastic deformation of the target in the microregions of the ion tracks [30,31,32,33,34]. It would be expected that ions of higher energy would produce higher surface deformations, since they lose energy at a high rate down to deeper target depths. The structure of the target material, and hence, its optical properties, are also changed by the energetic ion beam. But, as stated above, the optical path modulation caused by those changes was much smaller than that due to the surface modulation.

## 3. Results

### 3.1. Microscopic Study of the Gratings

The ion microbeam implanted gratings were studied first with a Zeiss Peraval transmission optical microscope (Oberkochen, Germany). The microscope was used in interference and interference phase contrast (INTERPHAKO) modes.

**Figure 5:**
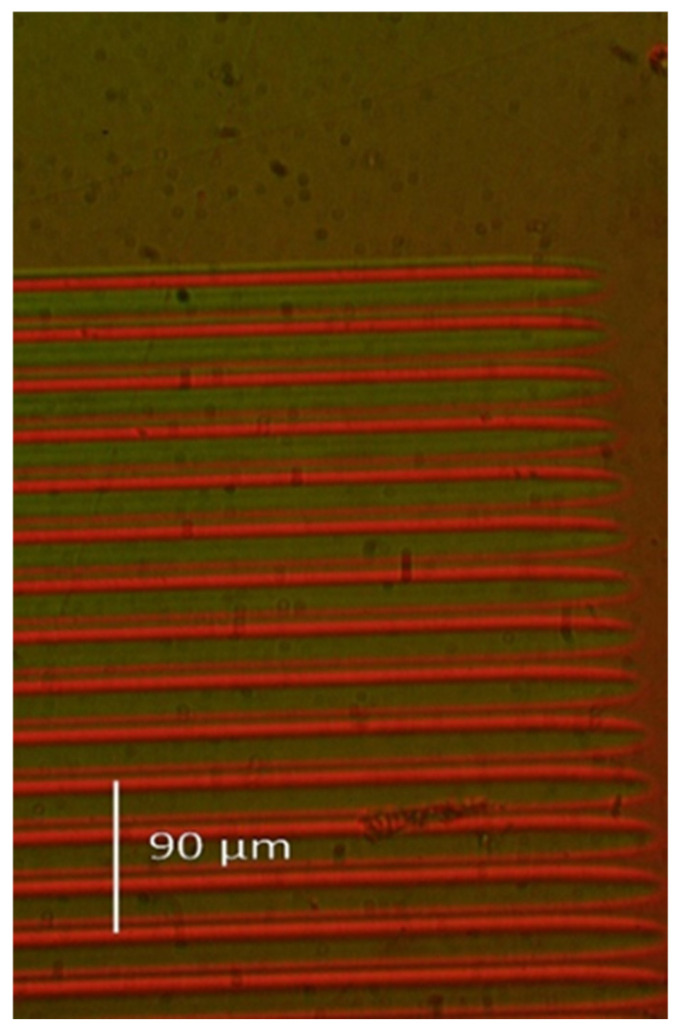
INTERPHAKO microphoto of a corner the sinusoidal grating GZ08, implanted in Bi_12_GeO_20_ by 10.5 MeV N^4+^ ions. F = 4 × 10^14^ ion/cm^2^. Λ = 16 μm.

**Figure 6:**
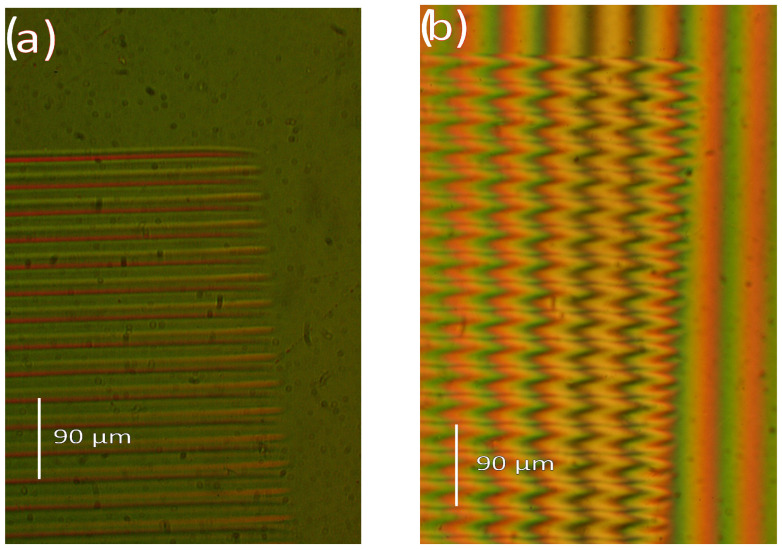
Microphoto of a corner of the blazed grating GZ06, implanted in Bi_12_GeO_20_ by 10.5 MeV N^4+^ ions. F = 4 × 10^14^ ion/cm^2^. Λ = 16 μm. (**a**) INTERPHAKO mode. (**b**) Interference microscopy mode.

**Figure 7:**
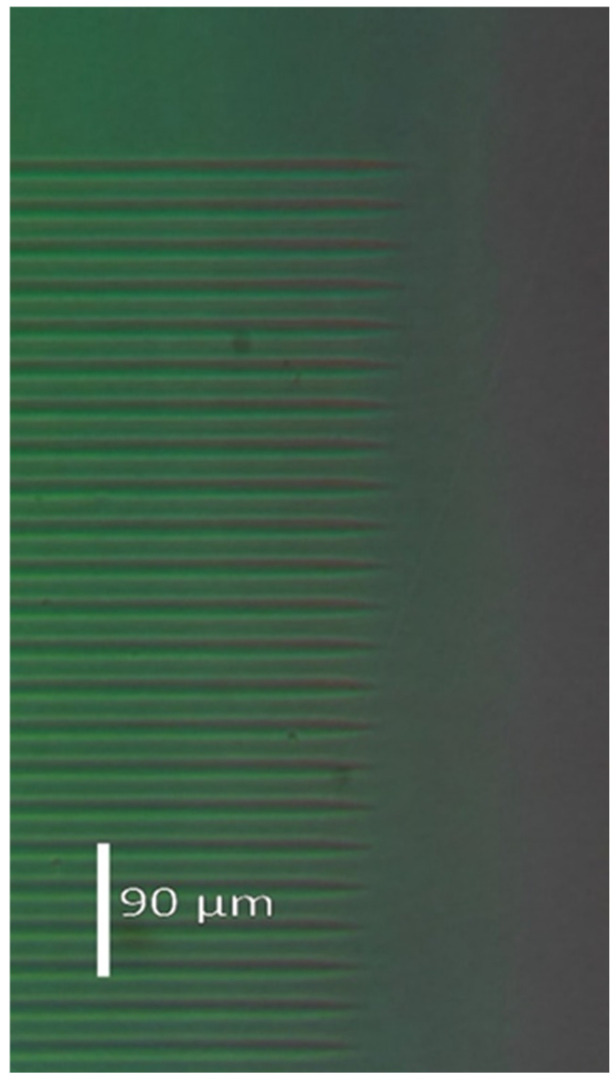
INTERPHAKO microphoto of a corner of the sinusoidal grating GL4, implanted in Er: Fe: LiNbO_3_ by 10.5 MeV N^4+^ ions. F = 1.4 × 10^15^ ion/cm^2^. Λ = 16 μm.

**Figure 8:**
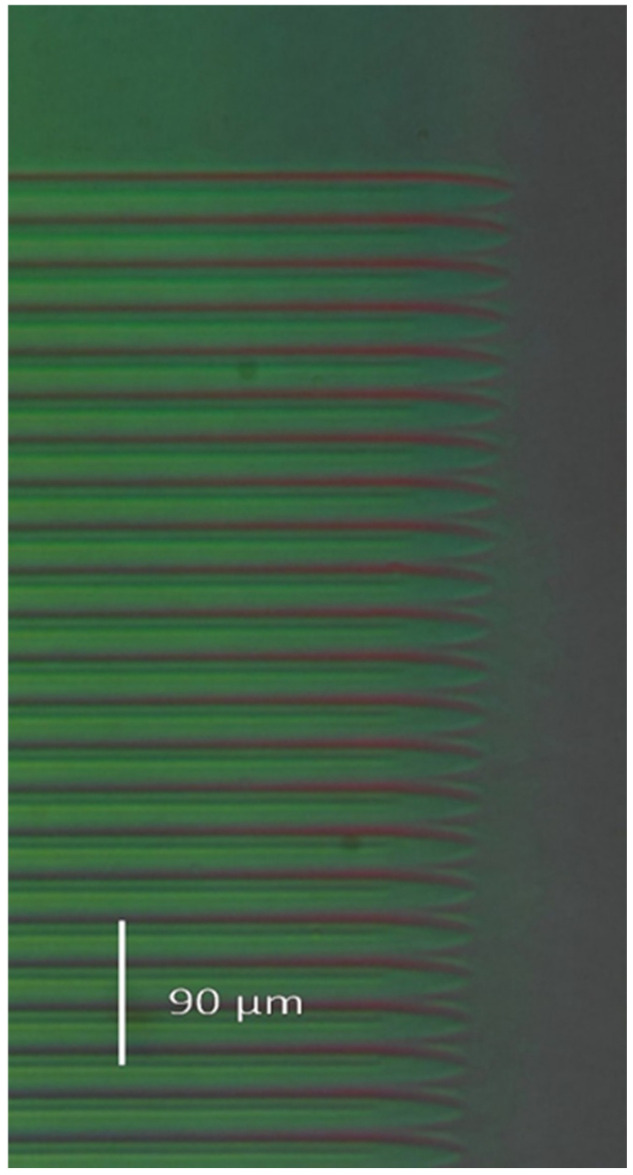
INTERPHAKO microphoto of a corner of the sinusoidal grating GL2, implanted in Er: Fe: LiNbO_3_ by 5 MeV N^3+^ ions. F = 5 × 10^15^ ion/cm^2^. Λ = 16 μm.

**Figure 9:**
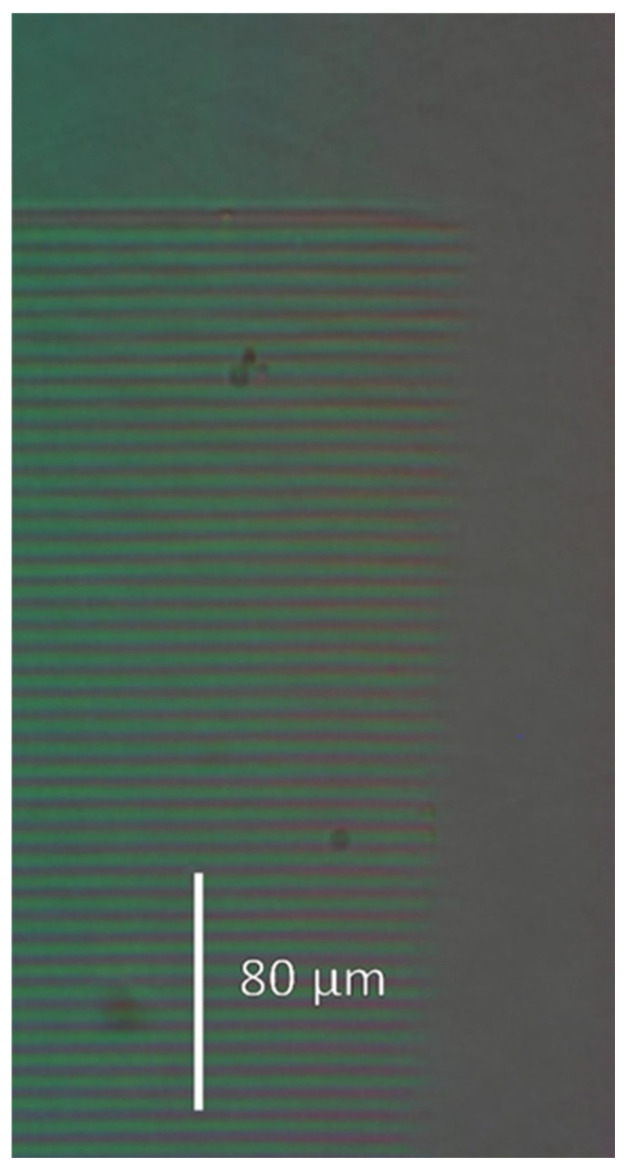
INTERPHAKO microphoto of a sinusoidal grating GL1, implanted in Er: Fe: LiNbO_3_ by 5 MeV N^4+^ ions. F = 5 × 10^15^ ion/cm^2^. Λ = 4 μm.

Since the ion microbeam implanted gratings are basically phase objects, ordinary light microscopy cannot be used to visualize them. Special techniques must be used. The first such technique is interference microscopy. Variations in the optical path across the microscopic image of the phase objects are transformed into deformations of a parallel equidistant interference fringe system by this method. The interference fringe system produced shearing interference between two identical images of the object. In INTERPHAKO mode, optical path variations are transformed into various interference colors. Representative microscopic photos of the ion microbeam implanted gratings are presented in Figure 5, Figure 6, Figure 7, Figure 8 and Figure 9.

Ion beam implanted gratings are mixed, i.e., both the index of refraction and the surface height are modulated. If either interference or INTERPHAKO microscopy is used in transmission mode, the total modulation of the optical path across the sample is shown, i.e., the algebraic sum of the index of refraction and surface height modulations. A possible method to separately determine the contributions to the total optical path modulation from the index of refraction modulation and the surface height modulation is the repetition of the microscopic measurements with and immersion-type objective. In such a way, surface relief modulation could be largely cancelled.

As the greatest part of the modulation (over 80%) of the optical path comes from the surface relief gratings in case of ion beam implanted gratings [22], we used optical microscopy only for qualitative assessment of the gratings. Exact profiles of the ion microbeam implanted surface relief gratings were determined by high-resolution optical surface profilometry.

The only difference in Figure 5 and Figure 6a is that the former is a sinusoidal grating, and the latter is a blazed one. The interference colors in Figure 5 range from green to crimson, indicating an amplitude of the modulation of the optical path over 200 nm. Figure 6b depicts the same blazed grating as Figure 6a but using interference microscopy instead of INTERPHAKO. In Figure 6b, the deformation of the interference fringes can be observed only over the grating, since the microscope slide did not introduce lateral variations in the optical path. It can clearly be seen in Figure 6b that there are sharp secondary peaks superposed on the expected triangular profile of the grating. The optical profilometric measurements confirmed this fact, as will be shown in Section 3.2.

INTERPHAKO microphotos of two sinusoidal grating recorded in Er: Fe: LiNbO_3_ crystal are shown in Figure 7 and Figure 8. Although the iron content of that crystal gives it a definite reddish color, thus impeding quantitative INTERPHAKO microscopy, one can clearly see the effect of the 3.6-fold fluence increase between the two gratings. The implanted regions are wider in the grating implanted at the higher fluence than in that implanted at the lower fluence. Moreover, a larger color contrast between the center and the edge of the grating lines can be observed in Figure 8, which is caused by the larger grating amplitude due to the 3.6-fold fluence. Finally, INTERPHAKO microphoto of the finest grating of a grating constant of 4 μm is shown in Figure 9. It shows a relatively high contrast and good uniformity.

### 3.2. Microprofilometric Study of the Gratings and Analysis of the Grating Profiles

**Figure 10:**
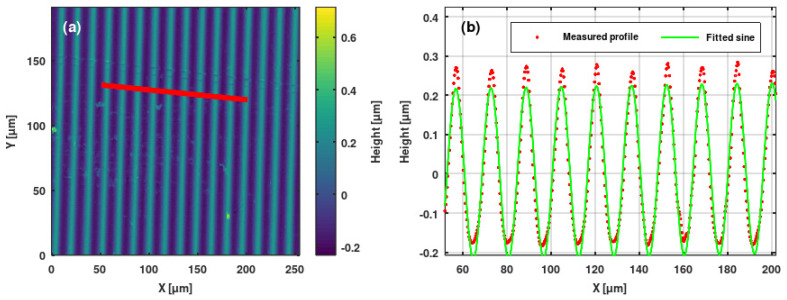
(**a**) Surface topography of the sinusoidal grating GH implanted in a Bi_12_GeO_20_ crystal. (**b**) Measured profile (red points) and fitted sine curve (green line) of same grating. The profile was extracted along the red line indicated in the topography.

**Figure 11:**
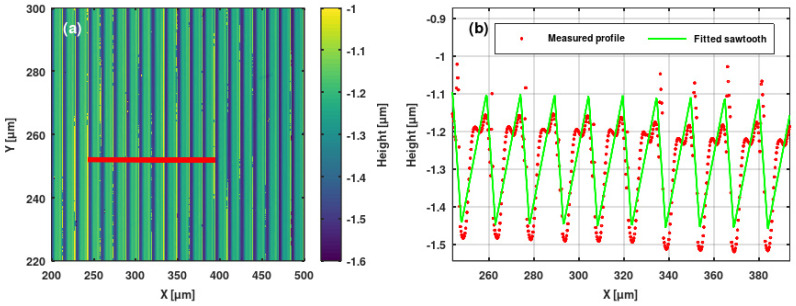
(**a**) Surface topography of the blazed grating GZ06 implanted in a Bi_12_GeO_20_ crystal. (**b**) Measured profile (red points) and fitted sawtooth curve (green line) of same grating. The profile was extracted along the red line indicated in the topography.

**Figure 12:**
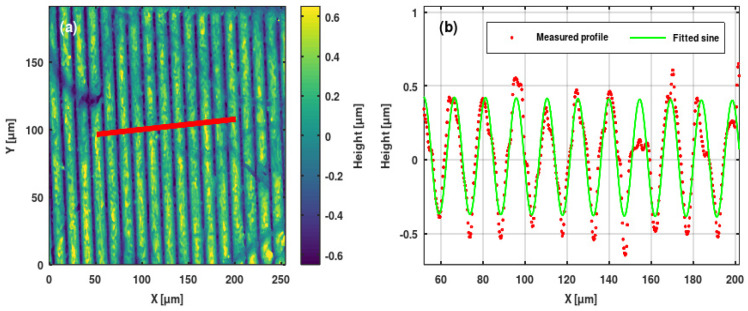
(**a**) Surface topography of the sinusoidal grating GL4 implanted in an Er: Fe: LiNbO_3_ crystal. (**b**) Measured profile (red points) and fitted sine curve (green line) of same grating. The profile was extracted along the red line indicated in the topography.

**Figure 13:**
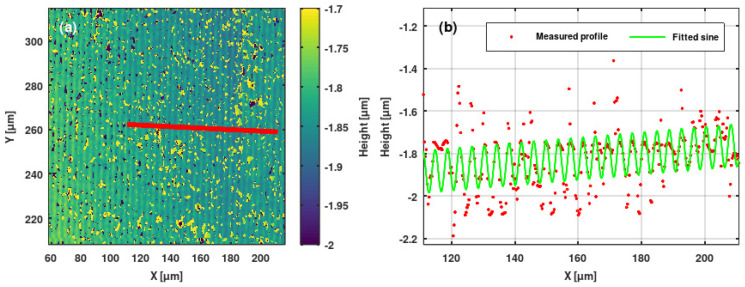
(**a**) Surface topography of the sinusoidal grating GL1 implanted in an Er: Fe: LiNbO_3_ crystal. Λ = 4 μm. (**b**) Measured profile (red points) and fitted sine curve (green line) of same grating. The profile was extracted along the red line indicated in the topography.

We used Sensofar PLU-2300 (Terrassa, Spain) [35] and Zygo NewView 7100 (Middlefield, CT, USA) [36] high-resolution optical profilometers to study the ion microbeam implanted surface relief gratings. We extracted grating profiles from the surface topographies. Sine and saw-tooth functions were fitted to the profiles. Results are shown in Figure 10, Figure 11, Figure 12 and Figure 13.

The profiles of the surface relief gratings were regular and, in general, smooth. In the greatest part of the gratings, sine and saw-tooth curves proved to be good to very good fits to the measured grating profiles.

Note that the profile of the blazed grating GZ06 has an amplitude of 350 nm, which is shown in Figure 11. It could be fitted well with a saw-tooth function, except for the appearance of two maxima in each line. The first one was like a shoulder, while the second one was needle-like. This phenomenon could be the attributed of the thermal and mechanical parameters of the Bi_12_GeO_20_ crystal, according to the above-mentioned thermal spike model.

As for the gratings implanted in Er: Fe: LiNbO_3_ and Er: LiNbO_3_ crystals, the surface of the implanted crystal became somewhat cracked, especially at higher fluences. Thus, the obtained grating profiles were not as smooth as for the Bi_12_GeO_20_ crystal (Figure 12).

The highest grating amplitudes were achieved in the doped LiNbO_3_ crystal, between 500 and 1600 nm. The surface topography of the finest grating is shown in Figure 13. Due to the very high implanted fluence of 5 × 10^15^ ion/cm^2^, the surface of the implanted region became irregular, such that the grating profile was not smooth. However, due to the high implanted fluence, the amplitude of the fitted sine curve was rather high at ~200 nm.

**Figure 14:**
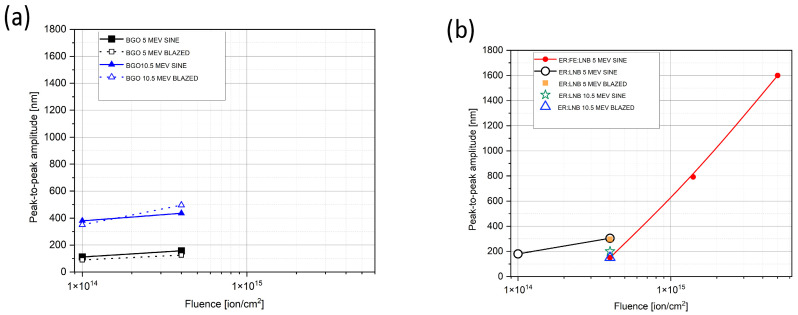
Fitted peak-to-peak amplitudes of the ion microbeam implanted gratings as a function of the implanted fluence in (**a**) Bi_12_GeO_20_ and (**b**) doped LiNbO_3_. Ion energy and grating type (sinusoidal or blazed) are parameters. Lines serve to connect the same group of gratings. Note that there are some overlapping points with two different symbols at fluences of 10^14^ and 1.4 × 10^14^ ion/cm^2^ and 1.4 × 10^14^ ion/cm^2^.

The fitted peak-to-peak amplitudes of all the ion microbeam implanted gratings are presented in Figure 14. The following conclusions can be drawn from that figure:

1. In these experiments, a higher fluence resulted in higher peak-to-peak grating amplitude, when the ion energy and grating type were the same.
2. Bi_12_GeO_20_ crystal implantations with the same fluence at an ion energy of 10.5 MeV produced much higher grating amplitudes than those performed at an ion energy of 5 MeV for both sinusoidal and blazed gratings. Namely, doubling the ion energy provided an increase of about 3.5 times in grating amplitude.
3. At an ion energy of 5 MeV, the same fluences produced 1.5–2 times larger grating amplitudes in the Er: LiNbO_3_ than in the Bi_12_GeO_20_. However, in the case of Er: LiNbO_3_ crystal targets, the increase of the ion energy from 5 MeV to 10.5 MeV did not produce an increase in grating amplitude. Instead, the amplitudes of both the sinusoidal and blazed gratings decreased considerably. But it must be stressed that there was only one value of fluence for which comparison could be made.
4. Further increasing the implanted fluence over 10^15^ ion/cm^2^ in the Er: Fe: LiNbO_3_ resulted in sinusoidal gratings of extremely large amplitude at an ion energy of 5 MeV. The peak-to-peak amplitude reached 800 nm for the GL4 grating, while surface damage was still minimal. Doubling the fluence produced a grating of an amplitude of 1600 nm, but with considerable surface damage.

### 3.3. Measurement and Calculation of the Diffraction Efficiencies

The diffraction efficiency of the ion microbeam implanted optical gratings was measured using a setup described in detail in our previous publication [24].

An SM600 Fabry-Perot pigtailed semiconductor laser (from Thorlabs, Newton, NJ, USA) was used in the experiments. It worked at a wavelength of 640 nm. A fiber optics collimator provided a free-space beam.

Ion beam implanted gratings were generally of 500 µm × 500 µm, and, in some cases, 1 mm × 1 mm lateral dimensions. The illuminating laser beam passed through an aperture of a diameter of 500 µm to ensure that no laser light bypassed the gratings.

Samples were mounted on a computer-controlled rotation stage. An auxiliary computer-controlled camera was used to locate the gratings on the sample and align the illuminating laser beam on them.

A Thorlabs PM100 detector was used to measure diffraction efficiencies between diffraction orders of −4 to 4. Efficiencies in both negative and positive orders were measured and averaged.

Measured first-order diffraction efficiencies of the ion microbeam implanted sinusoidal optical gratings are summarized in Table 2.

The highest diffraction efficiency grating in Bi_12_GeO_20_ crystal was GH of 0.15, while that in Er: LiNbO_3_ was GZ04 with a very high value of 0.25.

The measured first-order diffraction efficiencies of the ion microbeam implanted blazed optical gratings are summarized in Table 3.

The highest diffraction efficiency grating in Bi_12_GeO_20_ crystal was GZ06 of 0.236, while that in Er: LiNbO_3_ was GT with a very high value of 0.25.

The diffraction efficiency of thin sinusoidal gratings is given by the Raman-Nath equation [37,38]:

η*_m_* = J_m_ ^2^ (π Δnd)/λ (1)

where λ is the wavelength, d is the peak-to-peak amplitude of the grating, η*_m_* is diffraction efficiency in the m-th order, J_m_ is the m-order Bessel function, and Δn is the maximum difference of refractive index in the grating. For surface relief gratings,

Δn = n_grating_ − n_air_. (2)

**Figure 15:**
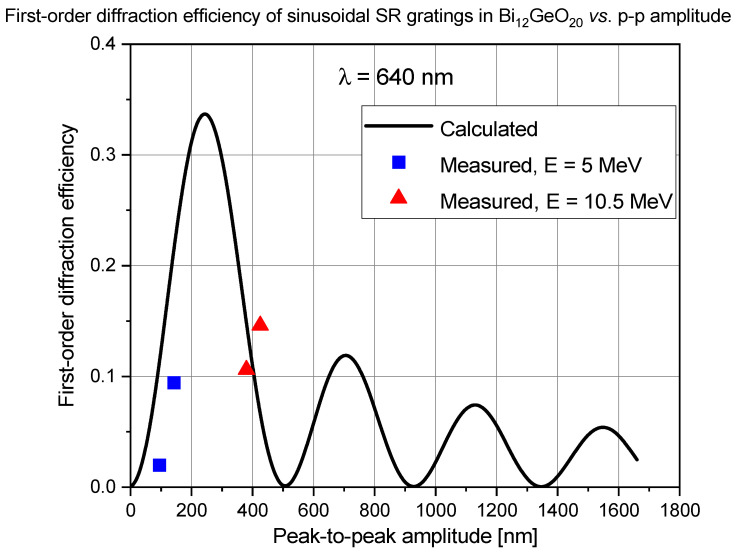
Calculated (line) and measured (points) first-order diffraction efficiency of ion microbeam implanted sinusoidal surface relief gratings in Bi_12_GeO_20_.

**Figure 16:**
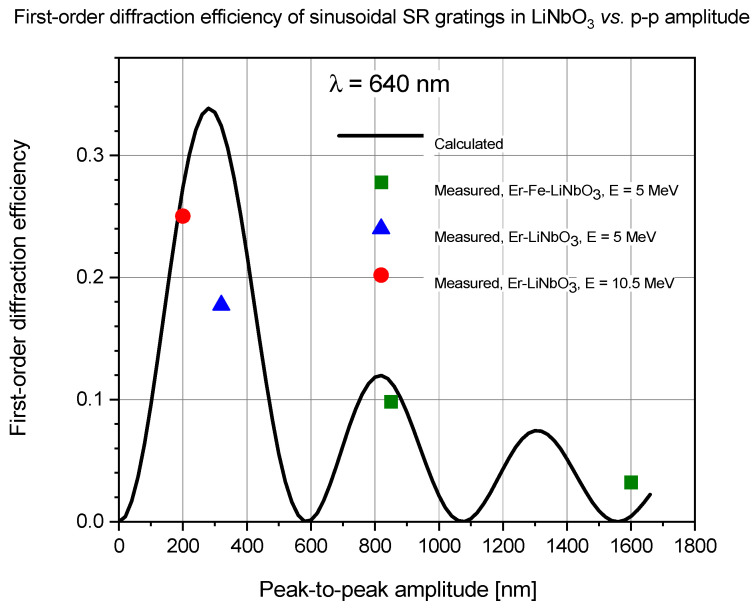
Calculated (line) and measured (points) first-order diffraction efficiency of ion microbeam implanted sinusoidal surface relief gratings in Er: Fe: LiNbO_3_ and Er: LiNbO_3_.

The maximum diffraction efficiency of a thin sinusoidal surface relief grating is 0.33 or 33%. To check whether the measured diffraction efficiencies are in accordance with the theory, first-order diffraction efficiency was calculated for sinusoidal gratings recorded in both materials and compared to the measured values of diffraction efficiency. Based on our above-mentioned previous experiences, we treated the gratings as pure surface relief gratings, that is, we considered the optical path length modulation introduced by the ion beam implantation induced refractive index changes to be negligible when compared to the optical path variations across the surface relief grating. Note that the highest refractive index modulations obtained under similar implantation parameters were in the 0.001–0.01 range. For the calculation of Δn, previously published values of the index of refraction of the crystal were used: for Bi_12_GeO_20_ n_grating_ = 2.55, and for LiNbO_3_ n_grating_ = 2.3. The results are presented in Figure 15 and Figure 16.

In the case of the BGO crystal, the measured diffracted efficiencies were well below the calculated values at 5 MeV. However, the two gratings implanted at an energy of 10.5 MeV showed only slightly lower or higher diffraction efficiency than that calculated (Figure 15).

In the case of Er: LiNbO_3_ crystals, the measured diffraction efficiency of the grating implanted with nitrogen ions of 5 MeV energy was also much lower than calculated. The grating implanted at an energy of 10.5 MeV energy had a measured diffraction efficiency very close to the predicted value, similar to the gratings implanted in the BGO crystal at the same energy (Figure 16).

In spite of their large amplitudes, the gratings recorded in Er: Fe: LiNbO_3_ had relatively low diffraction efficiencies (green squares in Figure 16). This was due to the overmodulation of those gratings. The useful range of the peak-to-peak amplitudes the gratings, where high diffraction efficiency can be achieved, was confined to the first narrow peak of the first-order Bessel function describing the diffraction efficiency. That range was between 200 and 400 nm. The second peak of the Bessel function, located at about 800 nm, attained only one third of the height of the first one. The third peak was located at 1300 nm, and its height was only one fifth of that of the first peak.

Besides of the possible errors in the measured grating amplitudes and diffraction efficiencies of the gratings, the difference between the measured and calculated diffraction efficiencies of the gratings could be attributed to the presence of strong higher harmonics in the grating profile, especially in the case of sinusoidal gratings implanted in BGO with 5 MeV nitrogen ion microbeam. The presence of strong harmonics manifested itself both in the relatively high higher-order diffraction efficiencies and was directly checked by Fourier analysis of the grating profiles.

The highest diffraction efficiencies of blazed gratings in both Bi_12_GeO_20_ and Er: LiNbO_3_ crystals were between 0.2 and 0.25. These rather high diffraction efficiencies were well below the theoretical maximum value of 1.0. This could be attributed to the fact that the diffraction efficiencies were measured at normal incidence, i.e., the angle of incidence was not optimized. Although the blaze angles of these ion implanted gratings were very low, i.e., between 0.5 and 2 degrees, they showed a very sharp peak in the diffraction efficiency vs. angle of incidence curve [39].

## 4. Discussion and Conclusions

A method was proposed and realized to fabricate surface-relief optical gratings of quasi-sinusoidal and saw-tooth profiles in optical crystals using microbeams of medium-mass ions in the 5–10.5 MeV energy range. Bi_12_GeO_20_, Er: Fe: LiNbO_3_ and Er: LiNbO_3_ crystals were used in the experiments. Nitrogen ion microbeams at energies of 5 and 10.5 MeV served to fabricate the gratings.

The measured profiles of the surface-relief gratings were fitted with sine or saw-tooth functions. The measured peak-to-peak amplitudes increased with the implanted fluence, reaching a maximum of about 1600 nm. For most of the gratings, the error of the amplitude of the fitted sine curve was only a few percent. This fact proved that it was indeed possible to fabricate surface-relief gratings with a quasi-sinusoidal profile using the microbeam based method. The highest first-order diffraction efficiencies achieved were ~25% in both the sinusoidal and blazed gratings. The latter grating type can yield much higher efficiencies when used in Littrow mount configuration.

The surfaces of the gratings written in Bi_12_GeO_20_ were generally smooth, even at implanted fluences above 5 × 10^14^ ion/cm^2^. However, the surfaces of the gratings implanted in Er: Fe: LiNbO_3_ crystals with fluences above 5 × 10^14^ ion/cm^2^ became grainy, and cracks even appeared. As for the gratings fabricated in Er: LiNbO_3_ crystals, the implanted fluence never exceeded 5 × 10^14^ ion/cm^2^ (see Table 1), and no grains or cracks could be observed on their surfaces. Therefore, it cannot be determined whether the excessive implanted fluence or the presence of the Fe dopant or both caused the degradation of the surface of the gratings. However, as one can see in Figure 16, the optimum values of peak-peak-to amplitude of sinusoidal surface relief gratings in LiNbO_3_ were between 200 and 400 nm. Grating GV had an ideal amplitude of 304 nm, and it was written at a moderate fluence of 4 × 10^14^ ion/cm^2^ (see Table 2). It is concluded that even rare-earth doped LiNbO_3_ can be effectively used for surface relief grating fabrication.

Some sinusoidal gratings with a grating constant of 4 μm were also successfully implanted by 5 MeV N^3+^ microbeam Er: Fe: LiNbO_3_ crystals, and their study by microscopy and optical surface profilometry showed that they possessed good sinusoidal profiles.

Grating couplers in biochemical sensors can be realized by the proposed method [40]. By using ion nanobeams instead of the microbeams, the minimum grating constant can be decreased to the range of 300 nm < Λ < 1000 nm. This way, it is possible to fabricate grating couplers working in the visible and near-infrared spectral bands. The same ion macro- micro and nanobeams could be used for the fabrication of other elements pertinent to biochemical sensors, e.g., planar and channel waveguides and micro interferometers [13].

The use of ion micro- and nanobeams for the direct writing of optical gratings onto crystals and glasses makes it possible to control the grating constant, grating profile, grating amplitude (in case of surface relief gratings), and the amplitude of the refractive index modulation (in case of index-of-refraction gratings) by carefully changing the implantation parameters. Furthermore, unlike other methods of grating fabrication, this is a single-step approach. The ion micro- or nanobeam implanted gratings, along with the planar and channel waveguides and microinterferometers fabricated by the same method, could be seamlessly integrated in optical biosensors, thus making it possible to fabricate monolithic devices.

## Figures and Tables

**Table 1:** Parameters of the ion microbeam implanted transmission optical gratings.

Name of the Grating Groups	Sample Material	Implanted Ion and Charge State	Ion Beam Energy [MeV]	Ion Beam Lateral Dimensions [μm × μm]	Current Density in the Microbeam [10^−3^ A/cm^2^]	Implanted Fluence [ion/cm^2^]	Grating Constant (Λ) [µm]	Grating Profile
GN	Bi_12_GeO_20_ crystal	N^3+^	5	4 × 4	0.70	4 × 10^14^	16	Saw-tooth
GP	Bi_12_GeO_20_ crystal	N^3+^	5	4 × 4	0.70	1 × 10^14^	16	Saw-tooth
GQ, GR	Bi_12_GeO_20_ crystal	N^3+^	5	4 × 4	0.70	4 × 10^14^	16	Sine
GS	Bi_12_GeO_20_ crystal	N^3+^	5	4 × 4	0.70	1 × 10^14^	16	Sine
GZ06	Bi_12_GeO_20_ crystal	N^4+^	10.5	4 × 4	1.0	4 × 10^14^	16	Saw-tooth
GZ07	Bi_12_GeO_20_ crystal	N^4+^	10.5	4 × 4	1.0	1 × 10^14^	16	Saw-tooth
GZ08	Bi_12_GeO_20_ crystal	N^4+^	10.5	4 × 4	1.25	4 × 10^14^	16	Sine
GZ09	Bi_12_GeO_20_ crystal	N^4+^	10.5	4 × 4	1.25	1 × 10^14^	16	Sine
GH	Bi_12_GeO_20_ crystal	N^4+^	10.5	2.5 × 10	0.57	4 × 10^14^	16	Sine
GL1	Er:Fe:LiNbO_3_ crystal	N^3+^	5	2.5 × 20	0.57	5 × 10^15^	4	Sine
GL2	Er:Fe:LiNbO_3_ crystal	N^3+^	5	3 × 20	0.57	5 × 10^15^	16	Sine
GL4	Er:Fe:LiNbO_3_ crystal	N^3+^	5	3 × 20	0.57	1.4 × 10^15^	16	Sine
GL6	Er:Fe:LiNbO_3_ crystal	N^3+^	5	3 × 20	0.57	4 × 10^14^	16	Sine
GT	Er:LiNbO_3_ crystal	N^3+^	5	4 × 4	0.57	4 × 10^14^	16	Saw-tooth
GU	Er:LiNbO_3_ crystal	N^3+^	5	4 × 4	0.57	1 × 10^14^	16	Saw-tooth
GV	Er:LiNbO_3_ crystal	N^3+^	5	4 × 4	0.57	4 × 10^14^	16	Sine
GW	Er:LiNbO_3_ crystal	N^3+^	5	4 × 4	0.57	1 × 10^14^	16	Sine
GZ02	Er:LiNbO_3_ crystal	N^4+^	10.5	4 × 4	1.0	4 × 10^14^	16	Saw-tooth
GZ03	Er:LiNbO_3_ crystal	N^4+^	10.5	4 × 4	1.0	1 × 10^14^	16	Saw-tooth
GZ04	Er:LiNbO_3_ crystal	N^4+^	10.5	4 × 4	1.0	4 × 10^14^	16	Sine
GZ05	Er:LiNbO_3_ crystal	N^4+^	10.5	4 × 4	1.0	1 × 10^14^	16	Sine

**Table 2:** Parameters and measured first-order diffraction efficiency of the ion microbeam implanted sinusoidal optical gratings. Missing values are due to problems in measuring the given quantity.

Name of the Grating	Name of the Sample	Implanted Ion	Energy of the Implanted Ion [MeV]	Implanted Fluence [ion/cm^2^]	Fitted Grating Amplitude [nm]	Measured First-Order Diffraction Efficiency
GS	Bi_12_GeO_20_	N^3+^	5	1 × 10^14^	112	0.02
GR	Bi_12_GeO_20_	N^3+^	5	4 × 10^14^	158	0.0937
GZ09	Bi_12_GeO_20_	N^4+^	10.5	1 × 10^14^	380	0.106
GH	Bi_12_GeO_20_	N^4+^	10.5	4 × 10^14^	436	0.1489
GL4	Er:Fe:LiNbO_3_	N^3+^	5	1.4 × 10^15^	792	0.098
GL2	Er:Fe:LiNbO_3_	N^3+^	5	5 × 10^15^	1600	0.032
GW	Er:LiNbO_3_	N^3+^	5	1 × 10^14^	180	
GV	Er:LiNbO_3_	N^3+^	5	4 × 10^14^	304	0.174
GZ05	Er:LiNbO_3_	N^4+^	10.5	1 × 10^14^		0.0073
GZ04	Er:LiNbO_3_	N^4+^	10.5	4 × 10^14^	200	0.250

**Table 3:** Parameters and measured first-order diffraction efficiency of the ion microbeam implanted blazed optical gratings. Missing values are due to problems in measuring the given quantity.

Name of the Grating	Name of the Sample	Implanted Ion	Energy of the Implanted Ion [MeV]	Implanted Fluence [ion/cm^2^]	Fitted Grating Amplitude [nm]	Measured First-Order Diffraction Efficiency
GP	Bi_12_GeO_20_	N^3+^	5	1 × 10^14^	90	0.0173
GN	Bi_12_GeO_20_	N^3+^	5	4 × 10^14^	126	0.0445
GZ07	Bi_12_GeO_20_	N^4+^	10.5	1 × 10^14^	350	0.138
GZ06	Bi_12_GeO_20_	N^4+^	10.5	4 × 10^14^	496	0.236
GU	Er:LiNbO_3_	N^3+^	5	1 × 10^14^		
GT	Er:LiNbO_3_	N^3+^	5	4 × 10^14^	296	0.250
GZ03	Er:LiNbO_3_	N^4+^	10.5	1 × 10^14^		0.0012
GZ02	Er:LiNbO_3_	N^4+^	10.5	4 × 10^14^	150	0.221

## Data Availability

Data are contained in the article.
